# Characterization and Discrimination of Apples by Flash GC E-Nose: Geographical Regions and Botanical Origins Studies in China

**DOI:** 10.3390/foods11111631

**Published:** 2022-05-31

**Authors:** Xinye Wu, Marie-Laure Fauconnier, Jinfeng Bi

**Affiliations:** 1 Institute of Food Science and Technology, Chinese Academy of Agricultural Sciences/Key Laboratory of Agro-Products Quality and Safety Control in Storage and Transport Process, Ministry of Agriculture and Rural Affairs, P.O. Box 5109, Beijing 100193, China; wuxinye@caas.cn; 2 Laboratory of Chemistry of Natural Molecules, Gembloux Agro-Bio Tech, University of Liege, Passage des Déportés, 2, 5030 Gembloux, Belgium; marie-laure.fauconnier@uliege.be

**Keywords:** apple, flash GC E-nose, volatile, multivariate analysis, discrimination, decision tree

## Abstract

Forty-one apple samples from 7 geographical regions and 3 botanical origins in China were investigated. A total of 29 volatile compounds have been identified by flash GC E-nose. They are 17 esters, 5 alcohols, 3 aldehydes, 1 ketone, and 3 others. A principal component analysis was employed to study the relationship between varieties and volatiles. A partial least squares discriminant analysis (PLS-DA), stepwise linear discriminant analysis (SLDA), and decision tree (DT) are used to discriminate apples from 4 geographical regions (34 apple samples) and 3 botanical origins (36 apple samples). The most influential markers identified by PLS-DA are 2-hexadecanone, methyl decanoate, tetradecanal, 1,8-cineole, hexyl 2-butenoate, (Z)-2-octenal, methyl 2-methylbutanoate, ethyl butyrate, dimethyl trisulfide, methyl formate, ethanol, S(-)2-methyl-1-butanol, ethyl acetate, pentyl acetate, butyl butanoate, butyl acetate, and ethyl octanoate. From the present work, SLDA reveals the best discrimination results in geographical regions and botanical origins, which are 88.2% and 88.9%, respectively. Although machine learning DT is attempted to classify apple samples, the results are not satisfactory.

## 1. Introduction

The apple is one of the most consumed and popular fruits in the food market worldwide, with both high nutritional values and a taste appreciated by a large number of consumers. Apples are rich in dietary fiber, sugars, minerals, and various bioactive components such as ascorbic acid (Vitamin C) and polyphenolic compounds [1]. Daily consumption of apples has been reported to potentially reduce the incidence of chronic non-communicable diseases (NCDs), namely, cancer, cardiovascular disease (CVD), and aging [2]. In 2020, the global production of apples was 86.44 million metric tons, ranking second after the production of bananas. Among them, China ranked first with 40.5 million metric tons, and the second to fifth were the United States of America, Turkey, Poland, and India (FAOSTAT, http://www.fao.org/faostat/en/, accessed on 19 April 2022). China’s apple production areas are mainly concentrated in the four major producing areas of the Bohai Bay region, the Loess Plateau in the northwest, the Old Road of the Yellow River, and the Cold and Cool Highlands in the southwest. Shaanxi, Shandong, Hebei, Shanxi, Liaoning, Henan, and Gansu Provinces are the seven major apple-producing provinces in China [3]. Based on their parents, apple cultivars in China could be classified into 4 cultivars, including cv. Fuji, cv. Delicious, cv. Golden Delicious, and cv. Ralls [4]. Aroma is an important indicator for evaluating apple quality and flavor. Different varieties or cultivars of apples present great differences in aroma compounds and content, and their composition and content can objectively reflect their flavor characteristics [5].

Although gas-chromatography mass spectrometry (GC-MS) is the most common method to study aroma/volatiles, attention to the electronic nose (E-nose) has been drawn increasingly due to the rapid analysis time and ease of operation [6,7]. Moreover, it has high sensitivity and a good correlation with the human sensory panel. E-nose is not only successfully employed in research laboratories but is used as a quality control tool in the industrial production stage as well [8]. A sensor-based E-nose could transform the sensor signal into a digital value, record data, and compute based on statistical models. A metal oxide sensor (MOS) is one of the most used sensors in this type of E-nose [9]. However, it could not reflect qualified or quantified results. The gas chromatography type E-nose (GC E-nose) is also named ultra-fast or flash gas chromatography, which is usually coupled with gas chromatography. Unlike sensor-based ones, flash GC (FGC) E-nose is possible to identify the volatile compounds [10,11].

From previous studies, E-nose has been widely applied in discrimination, shelf-life evaluation, authenticity assessment, and adulteration among different fruits, including apple, peach, tomato, mango, etc. In the case of apples, E-nose has been used in the areas of post-harvest treatments, shelf-life and maturity stage evaluation, and quality assessment [12]. For example, different types of E-noses have been applied in apple cultivar discrimination [13]. However, most of them were achieved by typical sensor-based E-nose. Although the application for FGC E-nose is not much of a sensor-based one, it had been successfully applied to the geographical origin discrimination in propolis [14], extra virgin olive oil [15], Chinese liquors [7] and cocoa liquors [16], botanical discrimination in pumpkin [17], adulteration in orange juice [18] and processing quality in jujube [9]. To the best of our knowledge, FGC E-nose has not been applied to apple geographical and/or botanical origin discrimination.

Principal component analysis (PCA), partial least squares discriminant analysis (PLS-DA), and stepwise linear discriminant analysis (SLDA) are commonly employed as multivariate analysis methods. They can be used to discriminate and classify apples [19,20,21] or apple products [22] by their volatile profiles successfully. Additionally, the application of machine learning to differentiate food samples has become more and more popular. The main machine learning methods include decision tree (DT), support vector machines (SVM), random forest (RF), etc. Aroma-related applications were mainly focused on the discrimination processing methods in strawberry juice [23,24], quality detection in citrus fruit [25], botanical origin discrimination in raw honey [26], classification in wines [27], and quality control in olive oils [28] by GC-MS, E-nose, E-tongue, and sensory evaluation.

In the present study, forty-one apple varieties have been investigated by FGC E-nose. The aims of the study were as follows: The first aim was to assess whether FGC E-nose could identify volatile compounds effectively. The second aim was to perform the classification of apple samples based on volatiles using the useful tool of multivariate analyses. The third aim was to attempt to apply machine learning methods to distinguish the apple’s geographical regions and botanical origins. Through the present study, we hope to provide new prospects for fruit sample discrimination and for protecting or authenticating agricultural products.

## 2. Materials and Methods

### 2.1. Apple Samples

In the present study, a total of 41 apple samples were collected from 7 geographical regions in China, they were Shandong, Shanxi, Sinkiang, Hebei, Gansu, Liaoning, and Shaanxi. Thirty-six of them belonged to the botanical origin of Golden Delicious (cv. GD), Fuji (cv. FJ), and Ralls (cv. RA). The rest of them were unknown. Detailed information was indicated in Table 1. All the samples were randomly collected from three apple trees with similar fruit weights and tree shapes. At the same time, all the apple samples were commercially mature and without any visible external damage, including decay, rot disease, and wormholes. After harvest, all the samples were transported to Institute of Food Science and Technology (Beijing, China) immediately and stored in a 4 °C refrigerator. When the apples reached the same stage of maturity, as determined by starch-iodine index [29], the analyses were performed.

### 2.2. FGC E-Nose

Prior to analysis, apples were picked from the 4 °C refrigerator, and stayed for 24 h at room temperature. After that, apples were cut into small pieces, and 5 g apple pieces were placed in 20 mL headspace vials and tightly capped with PTFE seals.

An FGC E-nose (Heracles II, Alpha M.O.S., Toulouse, France), connected with an auto-sampler (Odor Scanner HS 100, Alpha M.O.S., Toulouse, France), was employed in the present study. Moreover, the FGC E-nose was equipped with two parallel capillary columns and two flame ionization detectors (FIDs). The two columns are a non-polar MXT-5 (5% diphenyl and 95% methylpolysiloxane) and a slightly polar MXT-1701 (14% cyanopropylphenyl and 86% methylpolysiloxane). In order to achieve equilibration, the samples were incubated for 20 min at 50 °C. Afterward, 5000 μL was injected at 200 °C and 200 μL/s into GC system for 30 s. The initial and final trap temperatures were 15 °C and 240 °C, respectively. The trap procedure was maintained for 35 s. The vent of the trap was 10 mL/min. In the beginning, the oven was kept at 40 °C for 5 s and raised to 80 °C at the rate of 2 °C/s. Then, the temperature was increased to 230 °C (held for 20 s) with 1 °C/s. The temperature of the FIDs was 260 °C. Each sample was replicated 5 times.

### 2.3. Volatile Compounds Identification

A series of n-alkane (C6-C16) standard solutions (Sigma-Aldrich, St. Louis, MO, USA) was applied to calibrate volatile compounds under the same chromatographic conditions as described in 2.2. The Kovats retention indices (RI) were calculated based on the retention times. Then, the retention indices of identified volatile compounds were compared with AroChemBase (V6, Alpha M.O.S, Toulouse, France) library data and literature.

### 2.4. Data Processing

#### 2.4.1. Multivariate Analysis

The unsupervised technique PCA was extensively employed to visualize natural clustering in the data. In the present study, the variables were identified as volatile compounds by FGC E-nose, and the input values were the peak areas for each compound. The score and loading plots are used to demonstrate the differences/similarities between samples and explain the contribution to such differences/similarities.

PLS-DA and SLDA were all supervised pattern recognition models that distinguish samples into classes with prior knowledge. Apple samples were sorted into different groups based on their geographical regions and botanical origins. In the cases of geographical regions, because there was lack of representation for the samples in Hebei and Sinkiang, they were not considered. Four apple samples in Shanxi were also excluded since they came from the same place with different altitudes. Consequently, apple samples are grouped into 4 geographical regions (34 apple samples) and 3 botanical origins (36 apple samples).

PLS-DA was more commonly used to determine the features that best describe the differences among groups and which variables contribute more to classification. Variable importance in projection (VIP) was performed, and the volatile compounds with VIP ≥ 1 were considered the most influential markers in the extracted PLS-DA model [30].

SLDA is a robust statistical technique, which maximizes the variance between categories and minimizes the variance within categories. It provides a classification model by linear dependence of the classification scores in relation to the descriptors [24]. The original and leave-one-out cross-validation were used to identify and verify the model. F values in the program are set to enter and remove features in alternate steps to separate geographical regions and botanical origins based on the Wilks’ λ criterion [22].

The PCA and PLS-DA were analyzed by SIMCA (version 14.1, Umetrics, Sweden). The SLDA was performed by using SPSS (version 22, SPSS Inc., Chicago, IL, USA).

#### 2.4.2. Machine Learning

Decision tree (DT) is one of the most popular classification algorithms in current use in data mining and machine learning. It is a tress structure consisting of internal and external nodes connected by branches. Each internal node is associated with a decision function to determine which node to visit in the next step. Each external node indicates the output of a given input vector [31]. A classification and regression tree (CART), the most typical used method, was used in the present study.

The cross-validation technique is employed for increasing success of classifying algorithms and assessing the results objectively [32]. The V-fold cross-validation is one of the cross-validation methods, that is not only a useful tool for predictive data mining but provides simple models with optimal predictive capabilities as well [33]. In the present study, 5-fold validation was conducted to verify the models in DT. However, the number of samples was less than 5 in some geographical regions and botanical origins datasets, such as botanical cultivar of cv. Ralls and geographical region of Gansu. These parts of the data were replicated to achieve the minimum required by 5-fold validation. The final data sizes for geographical regions and botanical cultivars were 41 and 39, respectively. The average prediction accuracy was used to evaluate the models’ performance. This machine learning method was performed by Python package (version 3.8, Python Software Foundation, Wilmington, DE, USA).

## 3. Results

### 3.1. Volatile Identification

A total of 29 volatile compounds have been identified (based on the peak areas) among 41 apple samples. They were 17 esters, 5 alcohols, 3 aldehydes, 1 ketone, and 3 others. Identified compounds, retention indexes, and descriptive analysis results are presented in Table 2. The relative contents for each compound were calculated based on the peak areas, which may indicate the differences in the relative content of volatile compounds in the apple samples. The large standard deviations for each compound demonstrated that the apple samples were significantly different. The geographical regions and botanical origins were key factors that led to the differences. Eight volatile compounds could be found in all apple samples, which were isoamyl acetate, pentyl acetate, butyl butanoate, ethyl octanoate, hexyl 2-butenoate, octyl butanoate, ethyl undecanoate, and 2-hexadecanone.

Ethyl undecanoate was the highest one among all compounds, followed by hexyl 2-butenoate, isoamyl acetate, and butyl butanoate. They were the most dominant compounds in each apple variety. However, some compounds have been determined in a few apple varieties. For instance, 1-propanol, a primary volatile in the alcohol group [34], only existed in F7 and G6 and the peak areas for it were low. This was partly in agreement with Fellman et al. (2003) [35] that 1-propanol could not be detected in “Delicious” apples during the mature and storage stages. However, 1-propanol was the main compound in Starkrimson and Jonagold apples [36]. Similarly, ethanol, dimethyl trisulfide, (Z)-2-octenal, and methyl decanoate could only be identified in a few apple varieties. Notably, dimethyl trisulfide, (Z)-2-octenal, and methyl decanoate were mainly identified in Shandong, Shanxi, Gansu, and Sinkiang. Moreover, the compounds identified in the present study were not frequently identified compounds in apple, which had been reported only in seldom literature [20,37].

### 3.2. Multivariate Analysis

#### 3.2.1. PCA

**Figure 1:**
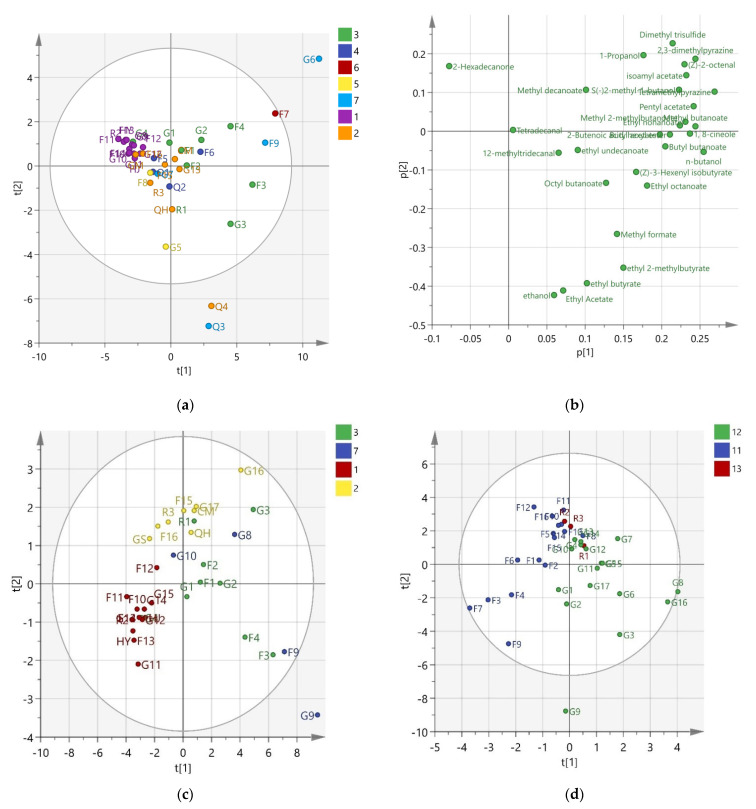

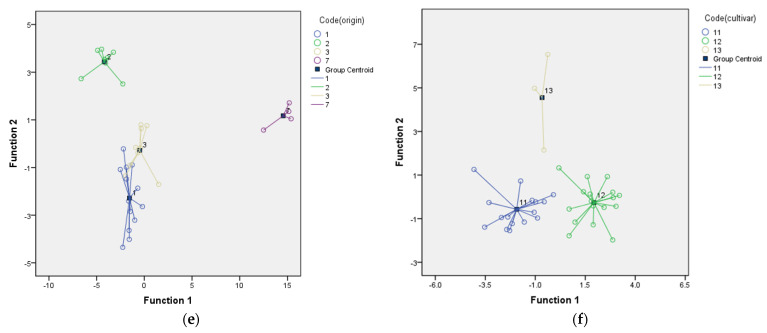
PCA, PLS-DA and SLDA plots. (**a**) PCA score plot; (**b**) PCA loading plot; (**c**) PLS-DA plot for geographical regions discrimination; (**d**) PLS-DA plot for botanical origins discrimination; (**e**) SLDA plot for geographical regions discrimination; (**f**) SLDA plot for botanical origins discrimination. In this figure, numbers from 1 to 7 were represented different geographical regions for apples, they were Liaoning, Shaanxi, Shandong, Shanxi, Hebei, Sinkiang, and Gansu, respectively. The numbers from 11 to 13 were represented different botanical origins of cv. Fuji, cv. Golden Delicious and cv. Ralls, respectively.

Six principal components (PCs) were obtained from the volatile compound data, which eigenvalues were higher than 1. The cumulative contribution was 86.7%. The first two PCs explained 58.2% of the total variance. The PC1 (43.5%) and PC2 (14.7%) were employed to draw PCA scores and loading plots (Figure 1a,b). The different colors represented different geographical regions.

It could be seen from the figures that the apple samples from Liaoning could be grouped together in the second quadrant. Combined with loading plots (Figure 1b), they were highly correlated with 2-hexadecanone. Except for Q4, other apple samples from Shaanxi and Shanxi were in the center of the original point, but they were mixed. Since these two places were located closely, they may be similar. In the case of other geographical regions, no clear separation could be observed. Furthermore, an obvious distinction could not be established based on the botanical origins.

Q3 and Q4 were located outside of the confidence level (95%) and in positive PC1 and negative PC2. From the loading plots, ethyl acetate, ethyl butyrate, and ethyl 2-methylbutyrate, located in the fourth quadrant and near the X-axis, were the main contributors in Q4. Q3 was mainly characterized by ethanol and ethyl acetate. They coincided with the relative percentages of peak areas (Supplementary Figure S1). Besides, G6 was dominated by 10 compounds, which were methyl butanoate, pentyl acetate, tetramethylpyrazine, 2,3-dimethylpryrazine, dimethyl trisulfide, isoamyl acetate, n-butanol, butyl butanoate, 1-propanol, and butyl acetate.

#### 3.2.2. PLS-DA

It can be seen from Figure 1c that 34 apple samples from 4 geographical regions were located differently. R2Y was used to evaluate the performance of the model corresponding to the goodness-of-fit and represents the variation of the Y that can be explained. The R2Y for the model of the geographical region was 0.666, and the model obtained a goodness-of-fit of 66.6%. Apples from Liaoning were mainly grouped in the third quadrant. The spots for Shaanxi apples were gathered above the X-axis and across the first and second quadrants. As for Shandong and Gansu, there was no clear separation, which appeared in a discrete state. The most important compounds determined by VIP values were 2-hexadecanone, methyl decanoate, tetradecanal, 1,8-cineole, hexyl 2-butenoate, (Z)-2-octenal, methyl 2-methylbutanoate, ethyl butyrate, dimethyl trisulfide, and methyl formate.

The discrimination model for 36 apples from 3 botanical origins was indicated in Figure 1d. In total, 66% of the goodness-of-fit for the botanical origins model was received. The cv. Fuji apples were on the left side of the Y-axis, whereas the cv. Golden Delicious was on the right. Three Ralls apples were distributed in the other two origins. The VIP values for ethanol, S(-)2-methyl-1-butanol, ethyl acetate, pentyl acetate, butyl butanoate, butyl acetate, methyl formate, (Z)-2-octenal, ethyl butyrate, and ethyl octanoate were greater than 1, which could be regarded as important compounds to discriminate samples.

#### 3.2.3. SLDA

A stepwise LDA (SLDA) was applied to visualize the classification of apple samples. In the case of geographical region discrimination, F values were set at 1.8 and 1.2 for including and removing from the model, respectively. Three canonical discriminant functions (DF) were used in the analysis, and 100% of the total variance could be explained. The first two DFs accounted for 80% and 13.1% of the total variance, respectively, which reached 93.1% of the cumulative variance. Fourteen variables were included by Wilks’ *λ* criterion. It could be seen from Table 3 that the total classification performance was 97.1% for the original sample groups and 88.2% for the cross-validation procedure. It should be noted that the performances for Liaoning, Shaanxi, and Gansu were very satisfactory because the percentages for the original group and cross-validation were all achieved at 100%. As for the apples from Shandong, the original correct percentage was 88.9%, whereas the cross-validated correct percentage was also low (55.6%). According to Figure 1e, some apple samples in Shandong (yellow label) were close to the apple samples in Liaoning (blue label).

At the same time, SLDA was also applied to classify the botanical origins of apples. For the variable selection, the usual probabilities for a variable included and removed were 1.3 and 0.5, respectively. The first two DFs explained 100% of the total variance, which were 64.4% and 35.6%, respectively. Eleven variables were included in the classification function coefficients. All the 36 apple samples were classified into the correct groups and separated obviously (Figure 1f). The total cross-validation percentage was 88.9%. Two apple samples from cv. Fuji were misclassified as cv. Golden Delicious and cv. Ralls. One apple sample from cv. Golden Delicious was regarded as cv. Ralls, and one sample from cv. Ralls was accounted as cv. Fuji.

### 3.3. Machine Learning

In order to explore the possibility of applying machine learning, a decision tree was attempted to discriminate between apple geographical regions and botanical origins. In total, 5-fold cross-validation was performed throughout the study. The average results were 76.07% and 64.64% for geographical regions and botanical origins, respectively.

**Figure 2:**
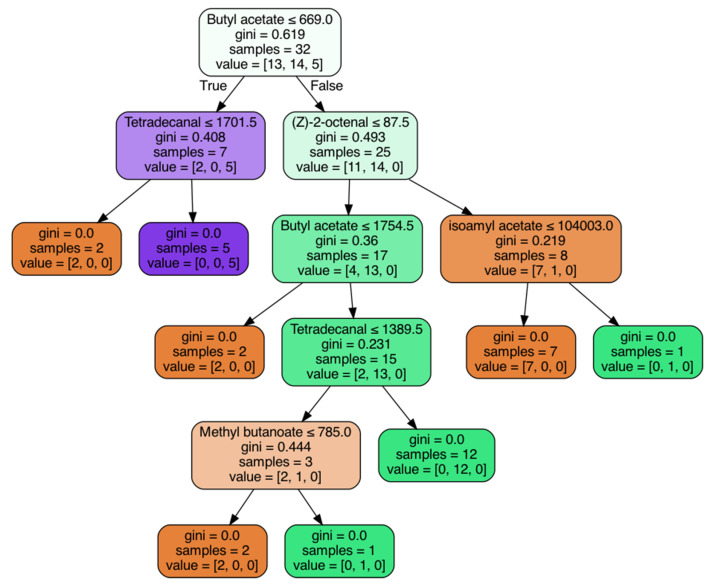
Decision tree diagram for a fold of the 5-fold validation of botanical origins. The white color was represented the whole sample. The orange, purple and green colors were represented cv. Fuji, cv. Ralls and cv. GD, respectively.

Figure 2 indicates a classification tree built by classification and regression trees (CART). Taking one-fold of the 5-fold validation model of botanical origins as an example, it could indicate which compounds played decisive factors in the classification. Notably, the sample size for this model was 32. Because the remaining 9 samples were used for validation. It could be seen from Figure 2 that butyl acetate, tetradecanal, (Z)-2-octenal, isoamyl acetate, and methyl butanoate were the main discriminating factors. Taking cv. Ralls (in purple color) as an example, it was mainly distinguished by butyl acetate and tetradecanal. When the peak area for butyl acetate and tetradecanal were lower than 669 and 1701.5, respectively, it could be recognized as cv. Ralls. In a similar way, volatile compounds that played a decisive role in distinguishing the other two cultivars could be found in Figure 2.

## 4. Discussion

It should be noted that some of the identified volatile compounds in the present study were inconsistent with the previous literature. Furthermore, some typical volatile compounds in apples, such as E-2-hexenal and hexanal, were not detected. The authors previously studied the volatile compounds of apples in Liaoning, China. Through headspace solid-phase microextraction (HS-SPME) GC-MS analysis, a total of 39 volatile compounds were identified [5]. By comparing the two studies, it could be found that only 7 compounds in this paper were consistent with the previous one. They were ethyl butyrate, butyl acetate, ethyl 2-methylbutyrate, pentyl acetate, butyl butanoate, ethyl octanoate, and hexyl 2-butenoate. Nevertheless, similar results were demonstrated in coffee aroma [38] and dried jujube fruit [9]. For instance, a total of 8 and 88 volatiles were identified by FGC E-nose and SPME GC-MS in coffee, respectively. Only one of the eight volatiles was detected by FGC E-nose and SPME-GC-MS. The remaining seven volatiles were unique to FGC E-nose [38]. In dried jujube fruit, the volatile compositions detected by FGC E-nose were also different from traditional SPME-GC-MS results [9]. One reason that caused volatile composition differences was probably due to the different columns applied between FGC E-nose and SPME-GC-MS. The non-polar (MXT-5) and slightly polar (MXT-1701) columns were equipped in the FGC E-nose, and they were less effective to detect polar compounds than the frequently used DB-WAX column [9]. Another reason might be that isolated and identified volatile compounds in FGC E-nose contributed to the smell of volatile compounds [38]. In qualitative analysis, the ability of FGC E-nose was not as satisfactory as SPME-GC-MS. However, compared with sensor-based E-nose, it can not only distinguish samples effectively but also obtain the specific compounds that lead to such results. If the equipment conditions are limited, FGC E-nose may be used for qualitative analysis, but the results have certain limitations. Consequently, compared to volatile identification, it is preferable to apply FGC E-nose for particular purposes such as geographical region and botanical origin discrimination.

Although PCA results could show differences and/or similarities in part, they did not reveal good separations among samples. Chinese researchers had obtained similar results. The volatiles of 50 apple varieties were analyzed. PCA results showed that the first two PCs explained 40.03% of the total variance, which was lower than in this study. Moreover, four cultivars (cv. Fuji, cv. Delicious, cv. Golden Delicious, and cv. Ralls) did not show clear separation either [4]. Further analysis should be conducted to achieve better discrimination of geographical regions and botanical origins. Therefore, PLS-DA, SLDA, and decision tree were applied in the present study.

To compare with PCA results, regardless of the geographical regions or botanical origins, better differentiation results could be seen in Figure 1c,d. However, it were worth noting that, no matter what kind of classification methods was used, the G9 (Huaniu, from Gansu) was away from other cultivars and lay outside the confidence interval. Huaniu apples are referred to, in particular to cv. Delicious apples are produced in Tianshui, Gansu Province. It was one of the three famous apple brands in the world that could be as famous as the Red Delicious in the USA and Fuji in Japan. It was the first apple variety in China to obtain an official trademark in the international market. In some literature on Huaniu apples, they are also shown differently from other apple cultivars. For example, Zou and Zhao (2008) [19] used a tin-oxide gas sensor array device and GC-MS to analyze three apple varieties’ (Fuji, Jina, and Huaniu) aroma volatiles and apply multivariate analysis to distinguish varieties. The number of aroma compounds in the Huaniu apple was less than that of the other two varieties. The esters in Huaniu were higher than them, such as ethyl propionate and butyl acetate. Consistent with the results of the present study, the sensory evaluation results revealed that it was very easy to discriminate Huaniu from the others. Because the aroma descriptions of these cultivars were different. Huaniu could be described as a “red apple aroma”, and the others could be described as a “sweet aroma” [19]. In addition, researchers compared different apple varieties to fermented cloudy apple juice (CAJ) and found the special aroma characteristics of Huaniu. The Huaniu CAJ had a high proportion of alcohols and esters and a relatively higher proportion of aldehydes. After fermentation, Huaniu fermented CAJ indicated a strong apple juice-like aroma due to its higher total soluble sugar content and lower organic acid content [39].

Through the analysis of PLS-DA, some geographical regions or botanical origins still cannot be effectively distinguished. Some data spots were overlapped on the figures (Figure 1c,d). To achieve a better discrimination result, the SLDA method was also attempted. Overall, the results of SLDA were better than those of PLS-DA.

The lower original and cross-validation rates mainly occurred in the analysis of Shandong apples. One apple sample from Shandong was misclassified into Liaoning when they conducted the original model. Nevertheless, two apple samples from Shandong were wrongly predicted to be Liaoning apples. They may have similar aroma profiles, leading to a decrease in model discrimination. The reason for this situation might be as follows: Shandong and Liaoning Provinces all belong to the Bohai Bay region. Shandong apple production accounted for 67.19% of the Bohai Bay region’s apple production, followed by Liaoning Province [40]. This demonstrated that apples from Shandong and Liaoning were absolutely dominant in the Bohai Bay region. Shandong and Liaoning are geographically close and belong to a temperate monsoon climate with relatively abundant and uniform precipitation. Although climate change may have effects on the volatile precursor formations, such as fatty acids and amino acids, thus impacting volatiles formation [5,41], Shandong and Liaoning Provinces share similar climatic conditions, such as precipitation, temperature, and light. Volatile differences due to climatic conditions were small compared to other geographical regions. Another two apple samples from Shandong were misclassified into Shaanxi. The possible reason may be that the latitudes of these two provinces are similar, basically between 35 and 38° N, thus sharing similar light conditions [42]. The length and intensity of light conditions may affect the quality of apples. There was little literature on the specific effect on the aroma. One research indicated that latitude was significantly negatively correlated with hexyl acetate concentration [43]. However, whether it was because of latitude that the apples in these two regions were not well-differentiated, it was necessary to further study the composition and concentration of their volatiles.

As for the SLDA results of botanical origins, the relatively low cross-validation percentage was mainly due to the apple cultivar resources. The cv. Fuji originated in Japan. It was a hybrid apple cultivar, and the female parent was cv. Ralls, and the male parent were cv. Golden Delicious [44]. These three cultivars may have similar characteristics, thus affecting the discrimination results. As a consequence, although 100% of cv. Fuji samples were correctly classified for the original groups, the cross-validation procedure was 87.5%. Similarly, this was why one sample from cv. Ralls was considered as cv. Fuji. Although there was only one sample that was classified incorrectly for cv. Ralls, the accuracy of the cross-validation was lowered because of the small sample size.

The prediction rates of the decision tree were not as good as expected, and they were lower than the SLDA results. Based on the existing data, there is a method that may improve the accuracy of the decision tree. It was to apply other decision tree methods, such as quick, unbiased, efficient regression tree (QUEST) and Chi-squared Automatic Interaction Detection (CHAID). Gagaoua et al. (2019) [45] used 3 decision tree methods (CART, QUEST, and CHAID) to predict beef tenderness and found that the 69.4% predictive accuracy of CHAID was the best decision tree method. Alternatively, other machine learning approaches, such as the support vector machine and random forest, could attempt to achieve better performance. However, whatever method is used, it is better to increase the sample size appropriately. The smaller sample size could easily lead to overfit or underfit, and the performance of the model will be worse. Whereas, the excessive sample size will also make the data more discrete, thereby reducing the predictive accuracy.

## 5. Conclusions

A total of 29 volatile compounds have been identified by flash GC E-nose from 41 apple varieties. Some of the identified volatile compounds were unusual in previous literature. Although the E-nose used in the study was the GC type, the ability to identify was not satisfactory. However, the results could be used to differentiate apple varieties. PCA results could show differences and/or similarities partly they did not reveal good separations among samples. Compared to PLS-DA and SLDA, the latter revealed the best performance in apple geographical regions and botanical origins discrimination and prediction among all the analyses. In addition to the commonly used multivariate analysis, the decision tree was also attempted to classify apple samples. However, the result was not as good as expected.

## Figures and Tables

**Table 1:** Information on apple cultivars collected.

No.	Samples	Origin	One Parent of Cultivar	Code	No	Samples	Origin	One Parent of Cultivar	Code
1	Starkrimon	Shandong	GOLDEN DELICIOUS	G1	22	Huafu	Liaoning	FUJI	F10
2	Fuji	Shandong (Xiqia city)	FUJI	F1	23	Huayue	Liaoning	-	HY
3	Ralls	Shandong	RALLS	R1	24	Huahong	Liaoning	GOLDEN DELICIOUS	G12
4	Starkrimon	Shandong (Taian City)	GOLDEN DELICIOUS	G2	25	Huajin	Liaoning	-	HJ
5	Fuji	Shandong (Taian City)	FUJI	F2	26	Ralls	Liaoning	RALLS	R2
6	Red General	Shandong	FUJI	F3	27	Hanfu	Liaoning	FUJI	F11
7	Golden Delicious	Shandong (Taian City)	GOLDEN DELICIOUS	G3	28	Hanfu	Liaoning (Xinmin City)	FUJI	F12
8	Golden Delicious	Shandong	GOLDEN DELICIOUS	G4	29	Starkrimon	Liaoning	GOLDEN DELICIOUS	G13
9	Yanfu 2	Shandong	FUJI	F4	30	Changhong	Liaoning	FUJI	F13
10	Fuji^1^	Shanxi	FUJI	F5	31	Qiujin	Liaoning	GOLDEN DELICIOUS	G14
11	Qinguan^1^	Shanxi	GOLDEN DELICIOUS	G5	32	Golden Delicious	Liaoning	GOLDEN DELICIOUS	G15
12	Fuji^2^	Shanxi	FUJI	F6	33	Nagafu 2	Liaoning	FUJI	F14
13	Qinguan^2^	Shanxi	GOLDEN DELICIOUS	G6	34	Ralls	Shaanxi	RALLS	R3
14	Fuji	Sinkiang	FUJI	F7	35	Fuji	Shaanxi	FUJI	F15
15	Fuji	Hebei	FUJI	F8	36	Ruiyang	Shaanxi	FUJI	F16
16	Wanglin	Hebei	GOLDEN DELICIOUS	G7	37	Qinguan	Shaanxi	GOLDEN DELICIOUS	G16
17	Fuji	Gansu	FUJI	F9	38	Qinhong	Shaanxi	-	QH
18	Qinguan	Gansu	GOLDEN DELICIOUS	G8	39	Changmiou	Shaanxi	-	CM
19	Huaniu	Gansu	GOLDEN DELICIOUS	G9	40	Granny Smith	Shaanxi	-	GS
20	Golden Delicious	Gansu	GOLDEN DELICIOUS	G10	41	Huangyuanshuai	Shaanxi	GOLDEN DELICIOUS	G17
21	Jonagold	Liaoning	GOLDEN DELICIOUS	G11					

**Table 2:** Identified volatile compounds in apple by flash GC E-nose.

	RI MXT-5	RI MXT-1701	*n*	Range	Minimum	Maximum	Mean	Std. Deviation
Methyl formate	384	464	41	2126.08	0	2126.08	860.16	701.33
Ethanol	423	560	41	1446.30	0	1446.30	164.89	341.73
1-Propanol	543	675	41	714.01	0	714.01	33.24	148.77
Ethyl Acetate	617	685	41	12,274.76	0	12,274.76	1003.79	2352.15
n-butanol	666	778	41	14,522.00	0	14,522.00	3306.51	3384.22
Methyl butanoate	716	786	41	9795.36	0	9795.36	961.91	1830.86
*S*(-)2-methyl-1-butanol	739	848	41	9514.74	0	9514.74	1808.47	2186.63
Methyl 2-methylbutanoate	776	851	41	2990.38	0	2990.38	316.37	643.46
Ethyl butyrate	802	865	41	77,811.04	0	77,811.04	8730.23	14,393.08
Butyl acetate	814	885	41	62,452.99	0	62,452.99	14,294.62	15,040.77
Ethyl 2-methylbutyrate	850	912	41	37,062.47	0	37,062.47	5655.66	8079.00
Isoamyl acetate	879	949	41	142,671.89	1181.87	143,853.76	25,960.22	29,205.89
Pentyl acetate	910	976	41	46,937.00	903	47,840.00	10,127.95	8520.48
2,3-dimethylpyrazine	944	1007	41	19,564.07	0	19,564.07	1872.54	4126.74
Dimethyl trisulfide	973	1041	41	1458.05	0	1458.05	103.66	288.68
Butyl butanoate	1000	1067	41	175,358.84	184.44	175,543.28	45,960.17	35,242.07
1,8-cineole	1043	1106	41	17,649.76	0	17,649.76	4307.92	4572.64
(*Z*)-2-octenal	1061	1128	41	1209.81	0	1209.81	132.91	320.17
Tetramethylpyrazine	1103	1174	41	16,011.48	0	16,011.48	3114.24	3453.25
(*Z*)-3-Hexenyl isobutyrate	1146	1212	41	3252.08	0	3252.08	942.02	862.89
Ethyl octanoate	1193	1263	41	32,805.69	1674.85	34,480.54	13,042.60	8268.36
Hexyl 2-butenoate	1240	1304	41	101,932.40	1378.28	103,310.68	30,478.65	26,044.90
Ethyl nonanoate	1287	1359	41	1577.50	0	1577.50	323.79	451.75
Methyl decanoate	1335	1403	41	937.35	0	937.35	69.85	200.44
Octyl butanoate	1388	1461	41	27,557.42	171.47	27,728.89	8605.66	5431.89
Ethyl undecanoate	1514	1559	41	255,585.74	12,471.57	268,057.31	84,013.89	53,310.55
12-methyltridecanal	1584	1658	41	3146.65	0	3146.65	850.73	787.85
Tetradecanal	1629	1687	41	4906.00	0	4906.00	2193.10	1153.65
2-Hexadecanone	1797	1901	41	3535.80	1840.79	5376.59	3004.46	827.97

**Table 3:** Classification results of SLDA.

		Original		Cross-Validated	
		Correct Number	Correct Percentage	Correct Number	Correct Percentage
Geographical regions	Liaoning	13/13	100%	13/13	100%
	Shaanxi	8/8	100%	8/8	100%
	Shandong	8/9	88.90%	5/9	55.60%
	Gansu	4/4	100%	4/4	100%
	Total	33/34	97.10%	30/34	88.20%
Botanical origins	cv. FJ	16/16	100%	14/16	87.50%
	cv. GD	17/17	100%	16/17	94.10%
	cv. RA	3/3	100%	2/3	66.70%
	Total	36/36	100%	32/36	88.90%

## Data Availability

The data presented in this study are available on request from the corresponding author.

## Supplementary Materials

The following supporting information can be downloaded at: https://www.mdpi.com/article/10.3390/foods11111631/s1. Figure S1. Heatmap displaying the percentage of 29 volatile compounds among 41 apple varieties. The color from red to green represented the percentages from low to high content for each variety. The cultivar acronyms are listed in Table 1.
