# Supplementary material for: Characterization and Discrimination of Apples by Flash GC E-Nose: Geographical Regions and Botanical Origins Studies in China
Source: Foods. 2022 May 31;11(11):1631. doi: 10.3390/foods11111631 (PMC9180093; doi:10.3390/foods11111631)
Supplement: Supplementary file 1 [file foods-11-01631-s001.zip › foods-1755375-supplementary.pdf]

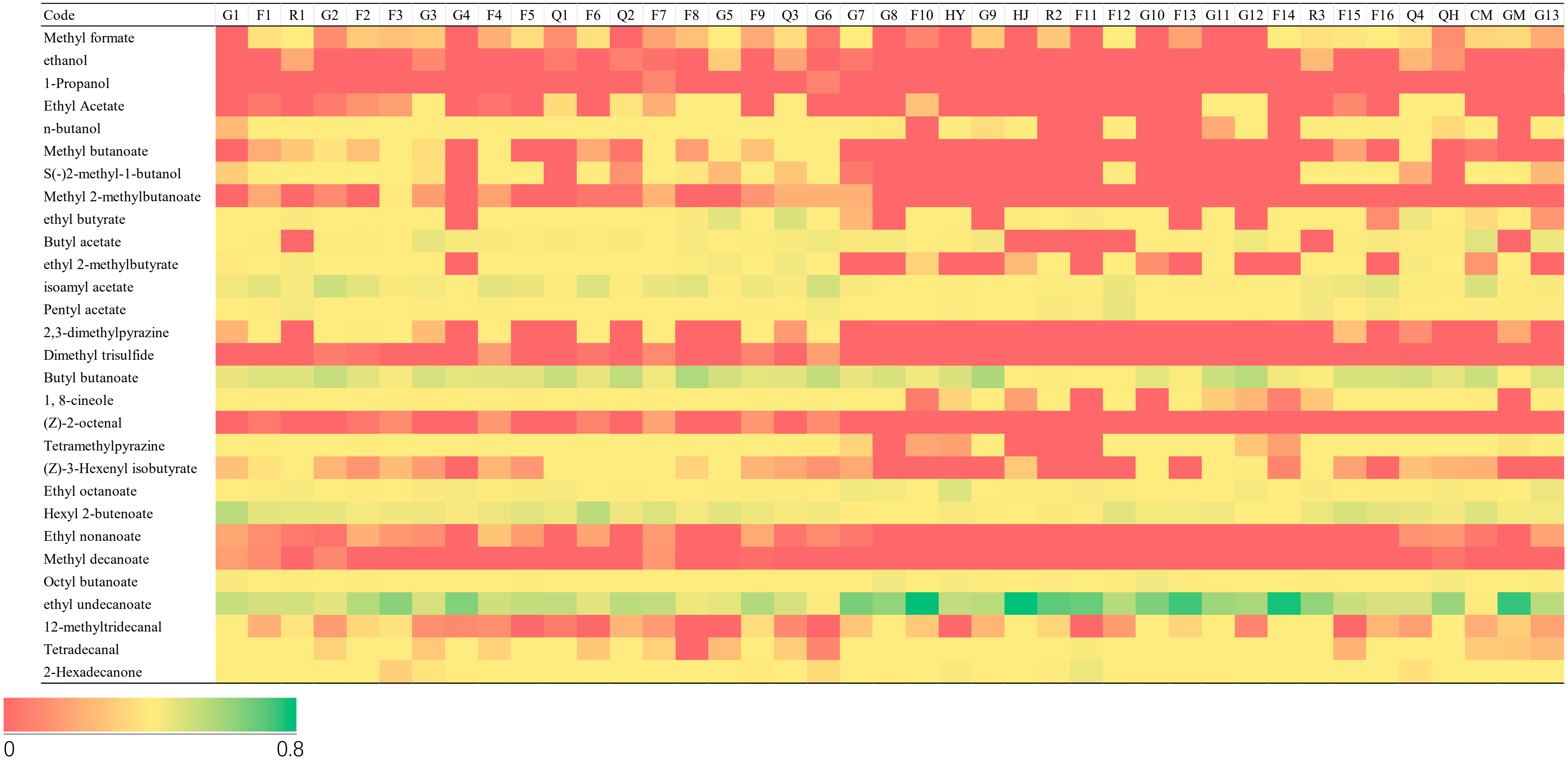

Figure S1. Heatmap displaying the percentage of 29 volatile compounds among 41 apple varieties. The color from red to green represented the percentages from low to high content for each variety. The cultivar acronyms are listed in Table 1.
